# Barrier‐forming, drug‐free nasal spray reduces allergic symptoms induced by house dust mite allergen

**DOI:** 10.1002/clt2.12277

**Published:** 2023-07-13

**Authors:** Patricia Couroux, Nicole Grosse, Anne Marie Salapatek, Yasmeen Goyal, Oliver Pfaar, Ilja P. Hohenfeld

**Affiliations:** ^1^ Inflamax Research DBA Cliantha Research Mississauga Ontario Canada; ^2^ Altamira Medica AG Zug Switzerland; ^3^ Department of Otorhinolaryngology, Head and Neck Surgery Section of Rhinology and Allergy University Hospital Marburg Philipps‐Universität Marburg Marburg Germany

**Keywords:** allergen exposure chamber (AEC), allergic rhinitis (AR), house dust mite (HDM), nasal spray, thixotropic formulation

## Abstract

**Background:**

House Dust Mite (HDM) is the most common indoor allergen triggering allergic symptoms. First‐line pharmacotherapy treatment is recommended in international guidelines, while the avoidance of allergens represents a still unmet guideline principle. AM‐301 is a new non‐pharmacological nasal spray that creates a protective gel‐like barrier on the nasal mucosa, preventing the contact with the allergens.

**Methods:**

This randomized, open‐label, 3‐period crossover study assessed the efficacy and safety of AM‐301. The objective was to determine whether AM‐301 reduces allergic rhinitis (AR) symptoms in patients exposed to HDM allergens. Adults with confirmed Perennial Allergic Rhinitis (PAR; *n* = 37) were exposed to HDM allergen in a controlled Allergen Exposure Chamber before and during a treatment course of AM‐301 (in six different sequences) within 3 weeks (A: One spray AM‐301 per nostril/B: Two sprays AM‐301 per nostril/C: no treatment). For the primary efficacy analysis, data from the total nasal symptom score (TNSS) were pooled from treatment A + B (D) and analyzed with Analysis of Covariance Model. As secondary endpoints, single time points, visits and symptoms were analyzed.

**Results:**

The primary endpoint (overall change in TNSS from baseline over all three visits) showed significant results (*p* = 0.0085). A comparable alleviation of all four symptoms (itchy nose, nasal congestion, runny nose, sneezing) by the protective layer started to emerge after 40 min and lasted up to 180 min (end of challenge). AM‐301 resulted to be safe and well‐tolerated.

**Conclusion:**

AM‐301 significantly reduced HDM‐related allergic symptoms in a standardized allergen challenge. Protection was observed to last up to 180 min.

## 1 INTRODUCTION

Allergic rhinitis (AR) is the most common atopic condition of the nasal mucosa, representing the second most frequent cause of chronic disease, with a prevalence of confirmed cases in adults from 17% to 28.5% in Europe.^1^, ^2^, ^3^ If AR is poorly controlled, it highly affects the quality of life, having a significant negative impact on general health, emotional well‐being, sleep, co‐morbidity illnesses and productivity.^3^, ^4^, ^5^, ^6^ Its main symptoms consist of nasal congestion, rhinorrhea, itchy nose and sneezing, triggered by exposure to indoor (i.e., House Dust Mite (HDM), animal dander, mold spores, etc.) and/or outdoor (i.e. pollen) environmental allergens. Since AR is not life‐threatening, most people affected by this condition tend to not seek medical advice until symptoms become intolerable.^7^ At that point, it has been reported that patients feel underserved by their attending physicians, often accompanied by a lack of diagnosis, undertreatment, or prescription of incorrect treatment.^8^, ^9^ In contrast, many reports such as “Allergic Rhinitis and its Impact on Asthma”, the ARIA guideline^10^ indicates that AR must be considered as a clinically relevant condition, contributing to the prolongation of viral infections of the respiratory tract and being closely linked to other inflammatory diseases that affect respiratory mucous membranes, such as asthma, rhinosinusitis, and allergic conjunctivitis.^11^, ^12^, ^13^

Since AR is a major risk factor for asthma exacerbation, specific guidelines focus on providing a global and pragmatic approach to the assessment and treatment of AR.^7^, ^8^ In order to achieve effective disease management, medications that can provide immediate and sustained symptom relief with minimal side effects are essential, since patients can experience nasal symptoms (e.g., sneezing, itching, rhinorrhoea, congestion) and non‐nasal symptoms (e.g., itching, tearing/watering and redness of eyes; itching of ears/palate; coughing) already within minutes of allergen exposure.^6^, ^9^ Available treatment options to control AR encompass (i) anti‐inflammatory pharmacotherapy, (ii) avoidance measures and iii) allergen‐immunotherapy (AIT), the latter being the only disease‐modifying option.^10^, ^11^, ^12^, ^13^

While total avoidance of inhalant allergens is not feasible in real life conditions, an increasing number of products seek to prevent physical contact between allergens and mucosal cells by forming a physical barrier within the nose. For example, cellulose powders demonstrated efficacy in patients with grass pollen related AR, mostly on days with lower pollen counts, and a pollen blocker cream was able to reduce total nasal symptom scores in patients diagnosed with perennial AR.^14^, ^15^

Intranasal cellulose powders have been reported to provide improvement of nasal symptoms in both children and adults comparable to intranasal corticosteroids.^16^, ^17^ These encouraging results pave the way for the use of nonpharmacological approaches in the management of AR.

The AM‐301 medical device (Bentrio®) is a thixotropic nasal spray that forms a gel‐like barrier on the mucosa. Treatment of AR is achieved by limiting the exposure of the nasal mucosa to airborne allergens, in this way preventing the pro‐inflammatory‐allergic cascade through for example, mast cells and cytokines.^18^, ^19^ Its key ingredient is bentonite, a natural mineral clay with important adsorbent, swelling and rheological properties, widely used as a pharmaceutical excipient.^20^ The product has recently been proven efficacious in reducing the viral titer in *ex vivo‐*human nasal epithelial cells^18^ thanks to its ability to create a mechanical barrier between the nasal mucosa and the external environment. The formulation is neither absorbed nor metabolized and does not have any pharmacological or immunological interaction with the human body and may therefore represent an alternative to the currently available pharmaceutical treatment options. Spray characterization provided information about the rheological properties of the spray, which becomes liquid after shaking and returns to a gel‐like state within the nasal cavity. A single spray of AM‐301 provided extended coverage of the nasal mucosa up to the inferior turbinates.^21^

In an earlier clinical study, AM‐301 was efficacious in reducing symptoms to at least the same extent as the comparator (cellulose powder) during controlled exposure to grass pollen in Allergen Exposure Chamber (AEC), while showing a faster onset of action.^22^

In the present study, the efficacy of the AM‐301 device in patients with another allergy (HDM‐related Perennial Allergic Rhinitis (PAR)) was also assessed in an AEC in a randomized, 3‐period crossover design. The prevalence of HDM allergy is also high with 60 to 130 million persons worldwide.^23^ Further secondary, exploratory objectives were assessed including the efficacy of one spray compared to no treatment, two sprays compared to no treatment, analysis of single time points, and analysis of individual nasal symptoms. In contrast to a traditional field allergen exposure study, this methodology allows a reproducible standardization of allergen exposure, temperature and humidity^24^ and therefore provides a prospective real‐time evaluation of the clinical benefit of intervention with AM‐301.^25^, ^26^

## 2 METHODS

### 2.1 Study design

This was a randomized, open‐label, 3‐period crossover study (ClinicalTrials.gov: NCT05122143). The study protocol and informed consent were approved by the Advarra institutional review board (IRB in ON, Canada) and written informed consent was obtained from all subjects prior to enrollment in the study. The study was conducted in accordance with the ethical principles of the Declaration of Helsinki, the International Council for Harmonization Good Clinical Practice guidelines, ISO 14155 requirements and all local regulatory requirements.

The study was conducted from November 10, 2021 (first subject first screening) to April 04, 2022 (last subject telephone call). The total study duration was approximately 65 days and consisted of 2 screening visits and 3 treatment visits (total 5 visits) followed by a telephone call. Patients with HDM allergy as assessed by their medical history and a positive skin prick test for Dermatophagoides pteronyssinus (der *p*) allergen were enrolled into the screening phase. Detailed inclusion/exclusion criteria are presented in Table S1. Subjects were randomized when they had shown an adequate level of AR symptoms during the screening HDM challenge (Visit 2), which was defined as a ≥4 point change in TNSS from pre‐challenge on at least two out of nine time points within the three hour challenges. At least 7 days after Visit 2 and Visit 3, eligible subjects were randomized to one of the six treatment sequences (ABC, BCA, CAB, ACB, BAC, CBA) at an equal ratio. The treatments were applied 10 min before the challenge at Visits 3, 4 and 5:

- **Treatment A**: One spray AM‐301 (0.14 mL) per nostril,
- **Treatment B**: Two sprays AM‐301 (0.28 mL) per nostril (at two defined spray angles in order to obtain a broader coverage of the nasal mucosa),
- **Treatment C**: no treatment.

The cross‐over design allowed treatment C to be used as internal control.

**FIGURE 1:**
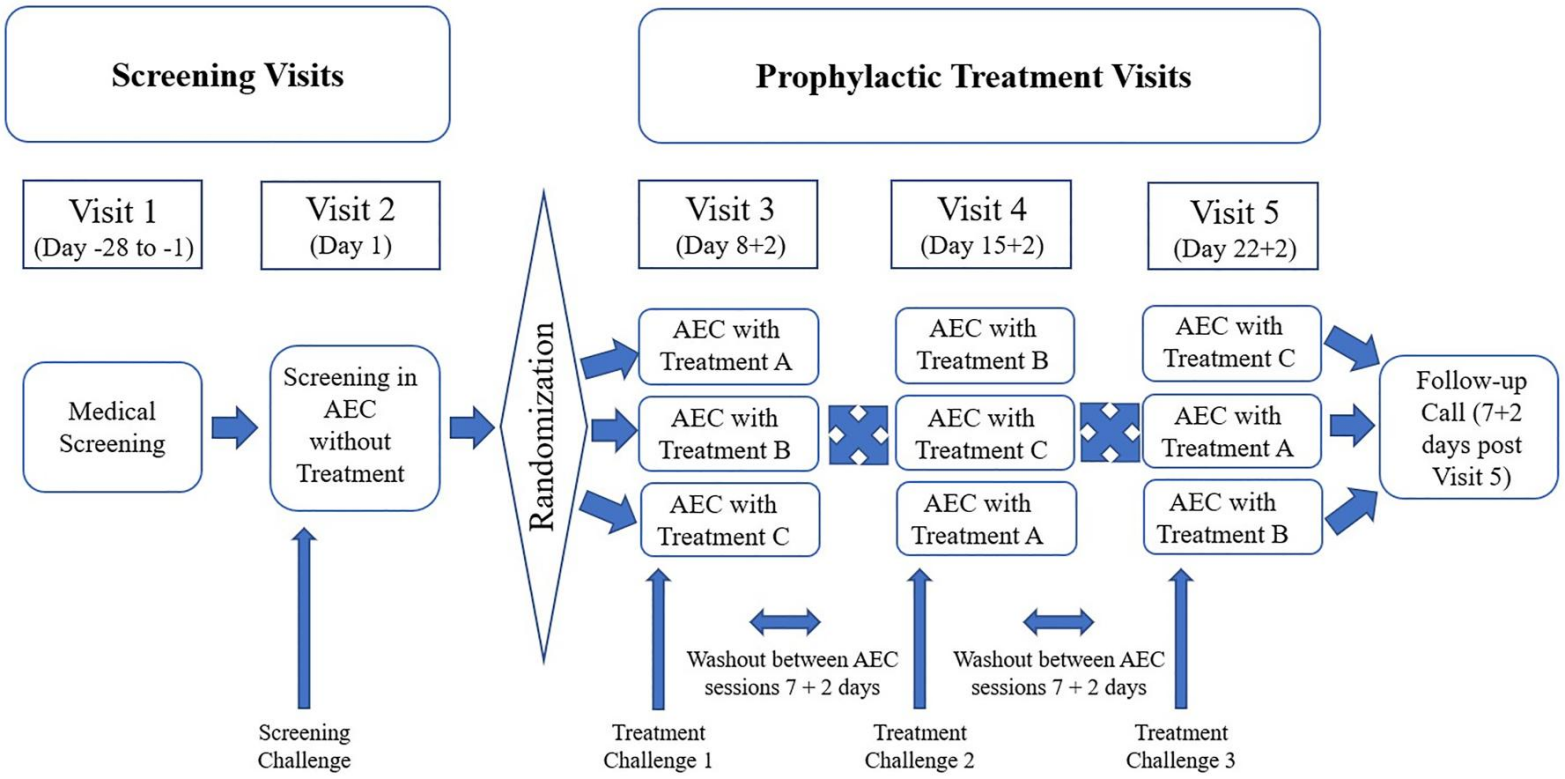
Study design. Visit 1: Medical screening; Visit 2: Screening in allergen exposure chamber (AEC); Visits 3, 4, and 5: prophylactic treatment visits in AEC (cross‐over sequences: ABC, BCA, CAB, ACB, CBA, BAC, each *n* = 6). Challenges lasted 3 hours with treatments 10 min prior to challenge. Treatments A and B, One spray and two sprays AM‐301 per nostril; Treatment C, no treatment.

**FIGURE 2:**
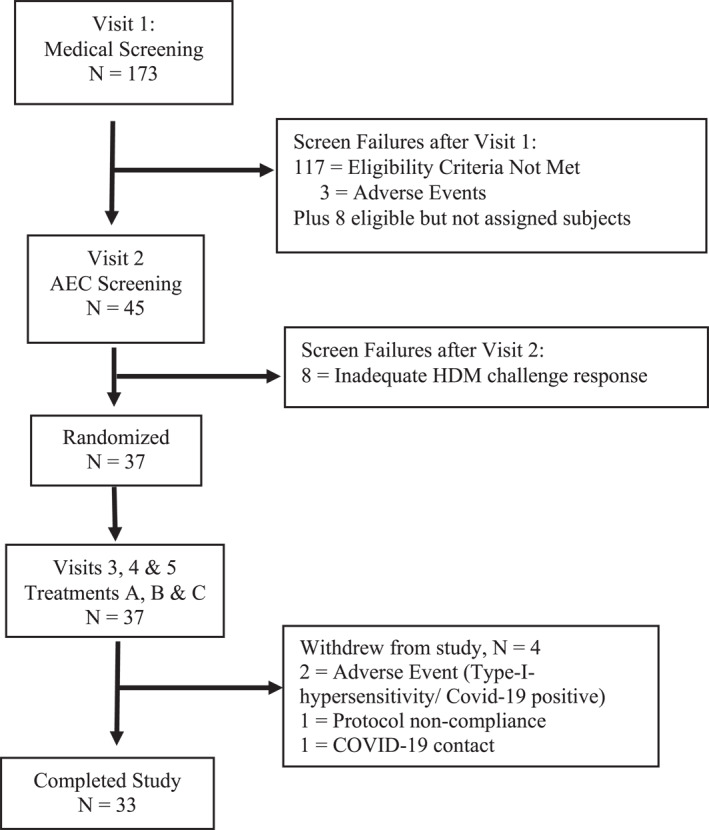
CONSORT diagram of the study.

The randomization schedule was generated using SAS (9.4 or higher) and was maintained under controlled access. The controlled 3‐h exposure to HDM allergen comprised dust mites (Dermatophagoides pteronyssinus) at a concentration of 20–100 ng/m^3^ aerosolized in the qualified AEC. In the chamber, each patient was asked to provide symptom scores every 20 ± 5 min. Between the three AEC treatment visits, there was a 7 ± 2 days wash‐out period (schematic study design: Figure 1 and CONSORT diagram: Figure 2).

### 2.2 Details on nasal spray

The investigational medical device, AM‐301 (Bentrio®, Altamira Medica Ltd., Zug, Switzerland), was manufactured by Laboratories Chemineau (France), following ISO 13485 and according to regulatory requirements (ingredients: purified water, glycerides, mixed decanoyl and octanoyl, propylene glycol, bentonite, glyceryl mono and dipalmitostearate, xanthan gum, disodium EDTA, mannitol, citric acid, butylated hydroxyanisole).

### 2.3 Assessments

#### 2.3.1 Efficacy assessment

Total nasal symptom score was chosen as the efficacy outcome measure, being an essential rating to assess the severity of symptoms in AR studies.^27^ The following nasal symptoms were scored by the study subjects: itchy nose, nasal congestion, runny nose and sneezing. The TNSS (score: 0–12) was calculated by summing the individual symptom scores for each symptom rated on a scale from 0 to 3, corresponding to: 0 = no symptoms, 1 = mild symptoms (sign/symptom clearly present, but easily tolerated), 2 = moderate symptoms (definite awareness of signs/symptoms that is bothersome but tolerable), 3 = severe symptoms (sign/symptom that is hard to tolerate and interferes with activities of daily living and/or sleeping). The study subjects recorded their symptom scores on an electronic patient reported outcome tablet (21CFR part 11 compliant system). In total, 10 time points (including pre‐challenge score) were collected during each AEC challenge.

#### 2.3.2 Safety assessment

Safety laboratory parameters were assessed during screening. Pre‐AEC subjects were monitored for vital signs by noninvasive blood pressure, heart rate recordings and respiratory rate measurements. At each visit, subjects reported any and all Adverse events (AEs), which were then assessed by a study physician as mild, moderate, or severe. In cases where concomitant medication was taken, clinical significance was judged by the investigator.

Adverse events were recorded throughout each visit. During a follow‐up telephone call after approximately 7 + 2 days of completion of Visit 5, subjects were asked for any occurrence of AEs after the last treatment and if they had taken any concomitant medications since then.

### 2.4 Study endpoints

#### 2.4.1 Efficacy endpoints

The overall change (average of all nine time points in the chamber over 3 hours) from baseline in mean TNSS for each treatment (A, B and C) at Visits 3, 4 and 5 (data of all three visits combined) was the primary efficacy endpoint. The following parameters were evaluated as secondary, exploratory efficacy endpoints: (i) The difference in TNSS at individual time points during HDM challenge between single and double application of AM‐301 and no treatment; (ii) The change from baseline of the individual nasal symptom scores at Visit 3, 4, and 5; (iii) The global rating for efficacy which was collected solely when subjects had received AM‐301, either single or double spray, during Visit 3, Visit 4 and/or Visit 5 reported by the study subjects and by the investigator as “very good”, “good”, “moderate” or “poor” (Questions: Tables S2a and S2b); (iv) to compare the efficacy of the AM‐301 device between treatment A and B.

#### 2.4.2 Safety endpoints

The occurrence of AEs and serious adverse events (SAEs) were analyzed. The global rating for tolerability was reported by the patients and the investigator at Visit 3, 4 and/or 5 at the end of the challenge only when they had received AM‐301 by answering a question with “very good”, “good”, “moderate” or “poor” (Tables S2a and S2b).

### 2.5 Statistical analysis

All efficacy analyses were performed on the intent‐to‐treat (Intention To Treat,ITT) population. The primary endpoint was also analyzed with the per protocol (PP) population. For the primary efficacy endpoint and exploratory secondary endpoints, the treatment comparison between treated and non‐treated subjects was performed using a mixed‐effects Analysis of Covariance Model (ANCOVA) for a 3‐period crossover study. The model included treatment, period (visit) and sequence as fixed effects, with baseline (pre‐dose) measurement as a covariate. Subjects nested within the treatment sequence were fitted as a random effect. Least square (LS) means and standard error (SE) by the treatment arm were extracted from the model and presented. Further, contrasts between LS means were extracted for:

- (1‐spray (A)+2‐sprays (B))/2 (D) versus no treatment (C);
- 1‐spray (A) versus no treatment (C), and
- 2‐sprays (B) versus no treatment (C).

The 95% confidence interval and 2‐sided *p*‐values are provided for each of these contrasts (LS means). All statistical analyses were conducted using SAS®, Version 9.4.

Adverse events were coded using the Medical Dictionary for Regulatory Authorities terminology version 24.1. The occurrence of AEs and SAEs were summarized in terms of incidence as well as in terms of the total number of AEs. Analysis of AEs in terms of incidence by severity and relatedness was provided.

### 2.6 Sample size calculation

It was hypothesized that the TNSS over 180 min after no treatment (group C) would be 2.3 units compared with 1.0 and 1.6 units for 2‐sprays (group B) and 1‐spray of AM‐301 (group A), respectively. Assuming that the standard deviation (SD) of the within subject change was 2 units, *N* = 30 randomized subjects provided 87.4% power at the 2‐sided 5% alpha level to test the hypothesis that the TNSS averaged across 1‐spray and 2‐sprays was superior to no treatment. Therefore, 36 subjects were randomized to ensure that at least 30 subjects completed the clinical investigation.

## 3 RESULTS

### 3.1 Study subjects

A total of 173 volunteers were screened for eligibility, and 37 study subjects (mean age 35.7 ± 10.5) randomized. Four subjects were withdrawn after randomization, which resulted in 33 subjects completing the study (details presented in CONSORT diagram, Figure 2). Two subjects stopped the study due to non‐device‐related AEs: Type I hypersensitivity and SARS‐CoV‐2 test positive. The study was conducted from Nov 2021 to Apr 2022. Both the safety (=full analysis set, FAS) and intent‐to‐treat (ITT) populations consist of 37 subjects and the PP population of 33 subjects. Demographic and baseline data per population are presented in Table 1. Demographics per sequence are presented in Table S3. The overall mean TNSS over the 180 min during screening differed with regard to severity between subjects: 19 subjects had on average mild symptoms (TNSS score <6), 14 showed on average moderate symptoms (TNSS score 6–9) and 4 subjects showed on average severe symptoms (TNSS score 9–12) (severity grading based on^28^, ^29^). The four subjects with mean severe symptoms were evenly distributed over 4 sequences. Sequences starting with treatment B (BCA, BAC) tended to have more mild cases overall and sequences starting with C (CBA, CAB) tended to have more moderate cases.

**TABLE 1:** Baseline characteristics of the study subjects according to the study population.

Study population	Safety/ITT (*N* = 37)	Per protocol *(N* = 33)
Age (Years), mean (SD)	35.7 (10.5)	36.9 (10.32)
Gender (Male/Female) *n* (%)	14/23 (38/62)	13/20 (39/61)
Race: Asian/Black/White/Other	6/6/24/1	6/6/20/1
Ethnicity: Hispanic or Latino	10	6
Height (cm) mean (SD)	168.6 (9.35)	169.2 (9.43)
Weight (kg) mean (SD)	72.0 (11.6)	72.4 (11.9)
BMI (kg/m^2^) mean (SD)	25.2 (3.0)	25.2 (3.0)

Abbreviations: BMI, Body Mass Index; ITT, Intention To Treat; SD, standard deviation.

### 3.2 Efficacy data

**FIGURE 3:**
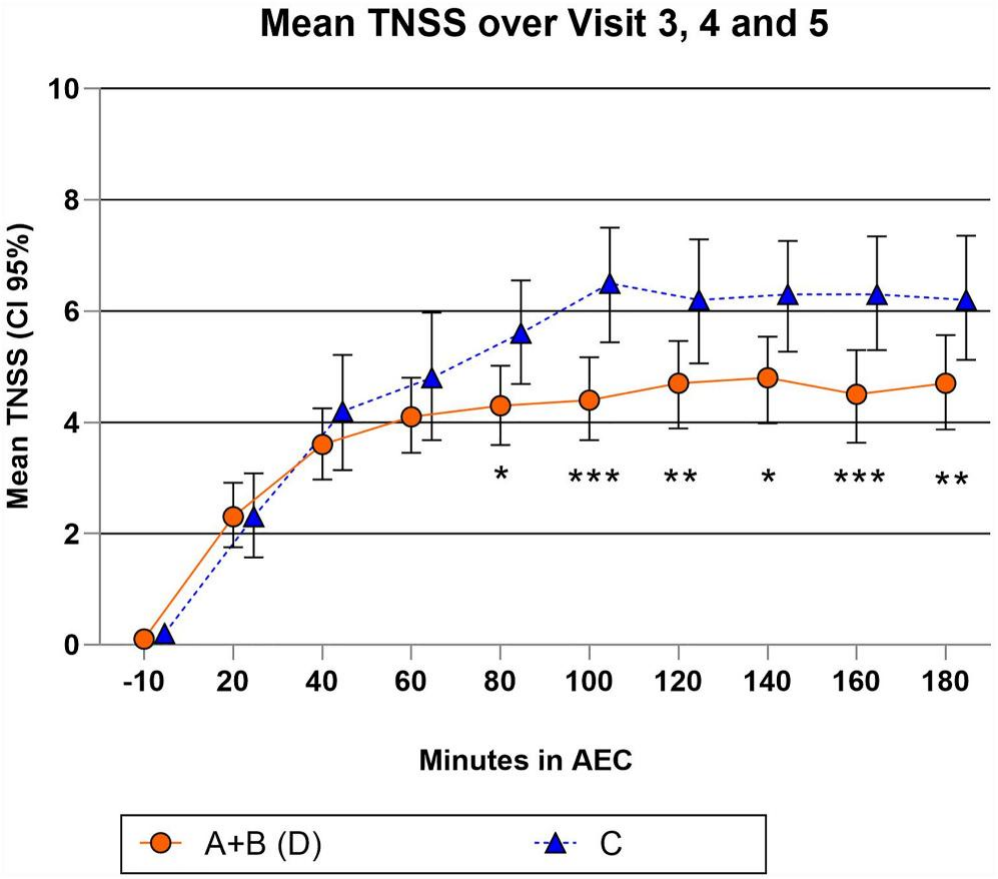
Graph with total nasal symptom score mean values of overall change in TNSS (0–180 min). Possible maximum score 12. Data from treatment visits 3–5 combined. Intention To Treat dataset. Device treatment A + B = treatment D. Treatment C = no device treatment. Mean ± CI 95%. CI, Confidence Interval; TNSS, Total Nasal Symptom Score; *p < 0.05, **p < 0.01, ***p < 0.001. *p*‐values result from the Least square Means per time point (refer to Table S5).

The primary objective was to compare the efficacy of the AM‐301 nasal spray (treatment D = A + B combined) and no treatment (C) in reducing nasal symptoms induced from HDM allergen exposure. The mean symptom score during the screening exposure was 5.95 ± 2.2 (*n* = 37). Mean symptom scores for device treatment D (A + B) and no treatment C combined from all three treatment visits were 4.2 ± 2.7 and 5.2 ± 2.6 (ITT), respectively (Table S4a) and 4.0 ± 2.8 and 4.9 ± 2.3 (PP). When data were analyzed with the prespecified ANCOVA model, there was a significant difference for the primary endpoint in the overall change in TNSS from baseline between AM‐301 versus no treatment (*p* = 0.009 (ITT) and *p* = 0.022 (PP)). Least square Mean (SE) was −1.08 (0.4) for ITT (Tables S4a and S4b). Mean symptom scores over 3 hours are presented in Figure 3.

The influence of the fixed effect parameters showed a significant impact of the visit number (*p* = 0.02) besides the treatment effect (*p* = 0.03) (Table S4c). However, treatment with AM‐301 showed a lower change from baseline in TNSS at post‐dose timepoints (0–180 min) than no treatment (C) at Visit 3, Visit 4, and Visit 5 with separation becoming apparent for 60 min (data not shown).

Secondary exploratory analysis of individual time points showed a significant difference for treatment D versus treatment C at all timepoints from 80 to 180 min (see Figure 3, Table S5).

**FIGURE 4:**
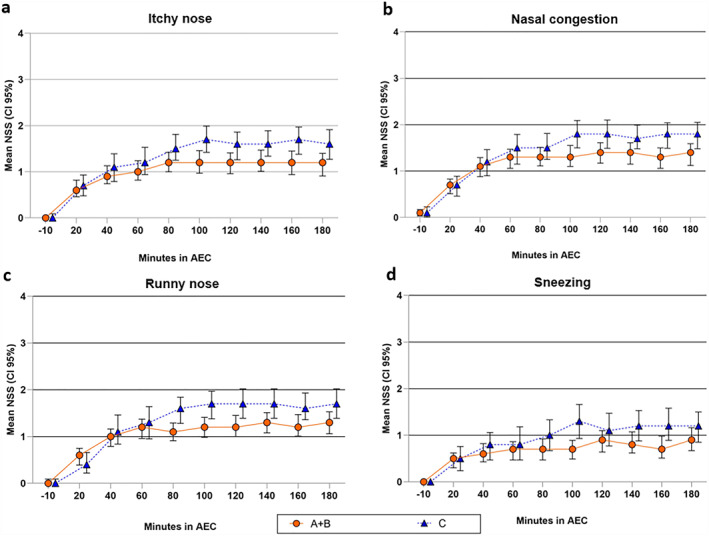
Graphical presentation of individual nasal symptom scores over time. A, Itchy nose, (B) Nasal congestion, (C) Runny nose, (D) Sneezing/Treatment A: One spray AM‐301 per nostril, Treatment B: Two sprays per nostril at different angles, D: Treatment A + B, Treatment C: no treatment = control; Data from visits 3–5 combined. Mean ± CI 95%. AEC, Allergen Exposure Chamber; NSS, Nasal Symptom Score.

An exploratory analysis of the four individual symptoms, namely itchy nose, nasal congestion, runny nose and sneezing, resulted in significantly lower scores for all four symptoms (ANCOVA: itchy nose *p* = 0.014; nasal congestion *p* = 0.038; runny nose *p* = 0.005; sneezing *p* = 0.028) when treatment D was compared to no treatment C (statistics Table S6; graphs Figure 4).

Global subjective efficacy of AM‐301 nasal spray was rated as “very good” or” good” by 61% of subjects for a single dose and 53% for the double dose. The investigator rated the efficacy as “very good” or “good” in approximately half of the subjects for the single and double doses of AM‐301 (Table 2A).

**TABLE 2:** Overall data for global efficacy (a) and global tolerability ratings (b). Global ratings were only collected after subjects have received AM‐301, either treatment A or B during Visit 3, 4 and/or 5.

2a				
Global efficacy rating response	Treatment A: AM‐301 ‐ 1 spray (*N* = 36)		Treatment B: AM‐301—2 sprays (*N* = 36)	
	n (%)		n (%)	
	Subject	Investigator	Subject	Investigator
Overall (data of visit 3, 4 and 5 combined)				
Very good	9 (25)	8 (22)	8 (22)	10 (28)
Good	13 (36)	10 (28)	11 (31)	9 (25)
Moderate	5 (14)	9 (25)	11 (31)	5 (14)
Poor	9 (25)	9 (25)	6 (16)	12 (33)

2b				
Global tolerability rating response	Treatment A: AM‐301 ‐ 1 spray (*N* = 36)		Treatment B: AM‐301—2 sprays (*N* = 36)	
	n (%)		n (%)	
	Subject	Investigator	Subject	Investigator
Overall (data of visit 3, 4 and 5 combined)				
Very good	17 (47)	22 (61)	20 (56)	20 (56)
Good	14 (39)	7 (19)	11 (30)	10 (28)
Moderate	3 (8)	3 (8)	3 (8)	3 (8)
Poor	2 (6)	4 (12)	2 (6)	3 (8)

The efficacy of one spray (treatment A) versus two sprays of AM‐301 (treatment B) was compared indirectly. Treatment A versus no treatment (C) resulted in significantly lower symptom scores when AM‐301 was used, *p* = 0.01 (ITT) and *p* = 0.02 (PP) (Table S4a+b). Treatment B versus no treatment (C) resulted as well in a significant difference over 180 min for the ITT dataset, *p* = 0.04, but not for the PP dataset, *p* = 0.099. Two sprays per nostril (treatment B) at different angles was efficacious in reducing symptoms but not to a greater extent than one spray per nostril (treatment A) with almost equal overall changes in mean TNSS of 4.1 ± 2.93 and 3.9 ± 2.59 for ITT population, respectively (Table S4a).

### 3.3 Safety data

A total of five AEs were reported in five subjects (Table S7). One AE (taste altered/unpleasant taste) in treatment group A was considered treatment‐related. It was mild in intensity and resolved after 30 min. No SAEs occurred.

Global subjective tolerability of AM‐301 was rated as “very good” or “good” by 86% of the subjects for both single and double doses. The Investigator rated the tolerability as “very good” or” good” in 80% of subjects for the single dose and 84% for the double dose (Table 2B).

## 4 DISCUSSION

This is the first clinical study assessing the efficacy of the AM‐301 nasal spray in patients allergic to HDM. In this open‐label, 3‐period crossover study, patients were randomized to receive AM‐301 ‐ one spray (treatment A), AM‐301 ‐ two sprays (treatment B) and no treatment (treatment C) in 3 standardized allergen challenges within 3 weeks. AM‐301 showed a significant reduction in mean nasal symptoms when compared to subjects who received no treatment in both primary and secondary endpoint analyses.

A recognized limitation of this study is that it was not placebo‐controlled and it was open‐label. Due to the galenic characteristics of AM‐301, a placebo control was not considered feasible as any formulation forming a gel on the mucosa would likely result in some kind of protection/therapeutic effect (active placebo). Even isotonic saline nasal sprays are known to sooth allergic symptoms,^30^ therefore excluding them as potential placebos. The study design was carefully considered. For ethical reasons, a study design was chosen that required the lowest possible number of patients to test three different treatments yet having sufficient statistical power for evaluating the primary endpoint.

Using a randomized and non‐fixed treatment sequence in a crossover design where all subjects had all treatments or no treatment meant that each subject served as their own control group, thereby reducing both intra‐ and inter‐subject variability. This is also the case for one or two sprays of treatment. Open‐label crossover trials are not uncommon. Blinding is often omitted in cases where the route of treatment is different between two treatment options or the packaging/texture of the drug/device cannot be made to look/taste/feel alike.^31^, ^32^, ^33^ A review of infliximab biological versus biosimilars analyzed 28 studies. Two double‐blind studies and 12 open‐label studies had baseline anti‐drug antibodies (ADAs) values. The comparison showed that ADA development and infusion reactions were similar between both study types. However, discontinuation rates were higher in open‐label trials.^34^ This points to the fact that expectations seem higher in an open‐label study. However, discontinuation rates also depend on the length of the study. Discontinuation rates in this study were low, irrespective of treatment sequence.

Notably, the findings suggest that subjects did not find additional benefit from 2 sprays versus 1 spray (further detailed below). The open design and therefore the subjects knowing their treatment with AM‐301 did not lead to a better outcome for the 2‐spray option.

Further, study conduct considerations included ensuring that the staff who were administering the treatment were different from the staff involved in monitoring the study participants, collecting subjective and objective data, and querying the study participants on AEs and thereby the latter staff were not aware of the subject's treatment regimen. As subjects knew their treatment, they were asked not to disclose it and to rate their symptoms at the time prompted or instantaneously.

As an efficacy measure, the subjects scored their nasal symptoms (TNSS), which is known to have a high reproducibility and sensitivity, making it a valuable outcome measure in AR studies.^27^ The score is used routinely in studies assessing symptoms/burden of AR sufferers and has shown clinical relevance.^35^ Especially in controlled allergen exposure studies, TNSS is used as the primary efficacy endpoint.^27^, ^36^

The study was powered to compare the overall change in TNSS between treatment A + B compared to no treatment C but not to test for each single time point or for treatment A or B alone. The symptom improvement was 1 and thus greater than the minimal clinically important difference of 0.55, which has previously been reported to be perceived by patients as a notable improvement and therefore being clinically relevant.^35^ A sensitivity analysis was performed excluding five subjects with major protocol deviations (1 x wrong treatment schedule assigned, 3 x premature study discontinuation due to AEs, 1 x noncompliance to study procedures). The symptom improvement was slightly reduced but still significant and clinically relevant (refer to Table S4b).

Another finding was, that the intensity of nasal symptoms was not only influenced by the treatment but also by the visit order. Indeed, TNSS showed decreasing ratings from Visit 3 to Visit 5 in all treatment groups, including the no treatment group C. Such a reduction in symptom score was also reported in the placebo group of another study with five HDM challenges over 6 months.^36^ A placebo effect cannot be the reason for this open‐label study using no placebo. Nevertheless, a difference between AM‐301 treatment and no treatment was still apparent at all visits.

When individual symptoms were assessed, namely itchy nose, nasal congestion, runny nose and sneezing, one or two sprays conferred similar protection compared with no treatment. AM‐301 improved all individual nasal symptom components. It is important to note that nasal congestion was also reduced, which is anti‐histamine resistant and is only treatable with corticosteroids. Nasal congestion is typically reported as one of the most “bothersome” symptom^3^ and is considered an underlying contributor to reduced quality of sleep and even related to higher absenteeism rates from work, particularly in PAR sufferers.^5^ These findings suggest a comprehensive effect of AM‐301 across major nasal symptoms. Those data support the hypothesis that allergens are “trapped” by the gel and prevent the initiation of the inflammatory cascade within the nasal mucosa.

Protection afforded by two sprays per nostril was comparable to one nasal spray per nostril. This finding is in line with data from a nasal residence time study with AM‐301. The coverage of the nasal mucosa and the nasal residence time with fluorescein‐labeled AM‐301 formulation was almost similar between one and two sprays per nostril.^21^ The application of a second spray at a different angle is not leading to a broader or longer coverage compared to when only one spray is administered per nostril.

The favorable safety and tolerability profile of AM‐301 reported before^21^, ^22^ was confirmed in this study. Both subjects and investigator rated global tolerability as “very good” or “good” in >80% of cases, irrespective of the number of sprays.

Based on the average effectiveness and nasal residence time of 3 h and the available safety information, the drug‐free barrier‐forming spray can be applied “as needed”, not more than every 3–4 h and 8 times per day. For example, patients who know they will be heavily exposed to dust mites in the next hours could make use of that drug‐free alternative treatment option.

In conclusion, this study has shown that the AM‐301 nasal spray device is clinically effective in alleviating symptoms after repeated AEC challenge in HDM‐related allergic patients. One spray per nostril provides adequate protection over a duration of 3 h. In addition, AM‐301 was found to be well‐tolerated without any relevant safety signals during treatment. These promising results should be confirmed in a larger field study under real life conditions.

## AUTHOR CONTRIBUTIONS

Study planning and design was a joint activity of Ilja P. Hohenfeld, Nicole Grosse, Patricia Couroux and Anne Marie Salapatek. Study management was performed by Nicole Grosse and study oversight was given by Patricia Couroux, Anne Marie Salapatek and Ilja P. Hohenfeld. Initial manuscript writing was done by Nicole Grosse and Ilja P. Hohenfeld with revision and major input by Ilja P. Hohenfeld, Anne Marie Salapatek, Patricia Couroux, Yasmeen Goyal and Oliver Pfaar. All authors have read and agreed to the published version of the manuscript.

## CONFLICT OF INTEREST STATEMENT

Nicole Grosse and Ilja P. Hohenfeld are employees of Altamira Medica AG and hold stock options. Altamira Medica AG has filed a patent application relating to AM‐301. Patricia Couroux, Anne Marie Salapatek and Yasmeen Goyal are employees of Inflamax Research DBA Cliantha Research that received funding from Altamira Medica AG for the conduct of this study. Oliver Pfaar reports personal fees from Altamira Medica AG during the conduct of the study. He reports grants and/or personal fees from ALK‐Abelló, Allergopharma, Stallergenes Greer, HAL Allergy Holding B.V./HAL Allergie GmbH, Bencard Allergie GmbH/Allergy Therapeutics, Lofarma, ASIT Biotech Tools S.A., Laboratorios LETI/LETI Pharma, GlaxoSmithKline, ROXALL Medizin, Novartis, Sanofi‐Aventis and Sanofi‐Genzyme, Med Update Europe GmbH, streamedup! GmbH, Pohl‐Boskamp, Inmunotek S.L., John Wiley and Sons, AS, Paul‐Martini‐Stiftung (PMS), Regeneron Pharmaceuticals Inc., RG Aerztefortbildung, Institut für Disease Management, Springer GmbH, AstraZeneca, IQVIA Commercial, Ingress Health, Wort&Bild Verlag, Verlag ME, Procter&Gamble, Meinhardt Congress GmbH, Deutsche Forschungsgemeinschaft, Thieme, Deutsche AllergieLiga e.V., AeDA, Alfried‐Krupp Krankenhaus, Red Maple Trials Inc., Technical University Dresden, ECM Expo& Conference Management, all outside the submitted work; and he is member of EAACI Excom, member of ext. board of directors DGAKI; coordinator, main‐ or co‐author of different position papers and guidelines in rhinology, allergology and allergen‐immunotherapy.

## INFORMED CONSENT STATEMENT

Informed consent was obtained from all subjects involved in the study.

## Supporting information

Supporting Information S1

## Data Availability

Research data are not shared.
