# Supplementary material for: Barrier‐forming, drug‐free nasal spray reduces allergic symptoms induced by house dust mite allergen
Source: Clin Transl Allergy. 2023 Jul 13;13(7):e12277. doi: 10.1002/clt2.12277 (PMC10345461; doi:10.1002/clt2.12277)
Supplement: Supplementary file 1 — Supporting Information S1 [file CLT2-13-e12277-s001.docx]

**TABLE S1**. Eligibility criteria for enrolment and randomization

| **Main subject selection criteria:** | |
| --- | --- |
| *Inclusion criteria* | *Exclusion criteria* |
| - >1 year diagnosis of Perennial Allergic Rhinitis to House Dust Mite - at Screening Visit 1 or within 12 months prior to the screening visit: positive Skin Prick Test (SPT) for Dermatophagoides pteronyssinus (der p) allergen - at Screening Visit 2: Total Nasal Symptom Score (TNSS) ≥ 4 over pre-AEC TNSS on at least two time points within the 3-hour HDM allergen challenge - at Screening Visit 1: spirometry assessment of FEV1 ≥80% of the predicted GLI-2012 value and a Tiffeneau ratio (FEV1/FVC ratio [FEV1%]) of ≥0.7 performed as per American Thoracic Society (ATS) standards - at Screening Visit 1: absence of pregnancy confirmed by pregnancy test or non-childbearing potential, no nursing or plan to become pregnant over the duration of the study - from at least 1 month prior to the Screening Visit 1 and until 72 hours after the last procedure: use of an effective method of birth control method - body mass index between 18.0 and 32.0 kg/m2 - from six hours before the allergen challenge during all EEC visits: smoke-free - agreement to abide by the study restrictions and comply with study procedures - absence of relationship dependency with the sponsor or study site staff at the time of informed consent and medical screening | - at the Screening Visit: clinically relevant nasal abnormality or trauma, abnormal clinical chemistry, haematology, urinalysis, vital signs, lung function, sensitivity AM-301 constituents - past or present: medical conditions which may either put the subject at risk for the participation in the study or may influence the results of the study - within 2 years prior to the study: use of medications considered to have an influence on the outcome of the study (i.e. allergen immunotherapy (AIT), local and systemic beta-blockers, ACE inhibitors, antipsychotic medications with antihistaminic effect, drug or alcohol abuse, etc) - within the 2 weeks prior to Screening Visit 1: SARS-CoV-2 infection - recent nasal ulcers, septum deviation or anything that can interfere with study results - any upper respiratory tract infection, asthma or condition that would prevent the subject from being able to stay for 3 hours in the chamber - within the 30 days prior enrolment: participation in another study - suspected inability to understand the protocol requirements |

**Table S2a**. Global rating of efficacy of the medical device and global rating of tolerability of the medical device by the subject

| “How would you describe the efficacy of the medical device in terms of improving your allergic symptoms compared to the first, unprotected stay in the pollen chamber?” | |
| --- | --- |
| Very Good | ❒ |
| Good | ❒ |
| Moderate | ❒ |
| Poor | ❒ |
|  | |
| “How would you describe the tolerability of the medical device?” | |
| Very Good | ❒ |
| Good | ❒ |
| Moderate | ❒ |
| Poor | ❒ |

**Table S2b**. Global rating of efficacy of the medical device and global rating of tolerability of the medical device by the investigator

| “How would you describe the efficacy of the medical device in the subject in terms of improving of allergic symptoms compared to the first, unprotected stay in the pollen chamber?” | |
| --- | --- |
| Very Good | ❒ |
| Good | ❒ |
| Moderate | ❒ |
| Poor | ❒ |
|  | |
| “How would you describe the tolerability of the medical device in the subject?” | |
| Very Good | ❒ |
| Good | ❒ |
| Moderate | ❒ |
| Poor | ❒ |

**TABLE S3.** Clinical characteristics of the study subjects, according to study sequence (Safety population).

|  | | | | | | | |
| --- | --- | --- | --- | --- | --- | --- | --- |
|  | ABC N=6 | BCA N=6 | CAB N=6 | ACB N=6 | BAC N=7 | CBA N=6 | Total N=37 |
| Sex (n (%)) |  |  |  |  |  |  |  |
| Male | 0 (0) | 3 (50.00) | 3 (50.00) | 4 (66.67) | 1 (14.29) | 3 (50.00) | 14 (37.84) |
| Female | 6 (100) | 3 (50.00) | 3 (50.00) | 2 (33.33) | 6 (85.71) | 3 (50.00) | 23 (62.16) |
| Age (Completed years) |  |  |  |  |  |  |  |
| n | 6 | 6 | 6 | 6 | 7 | 6 | 37 |
| Mean (SD) | 33.5 (8.34) | 44.8 (14.44) | 28.3 (4.84) | 40.7 (9.54) | 35.4 (10.45) | 31.7 (6.74) | 35.7 (10.47) |
| Median | 34.0 | 46.0 | 27.0 | 38.5 | 36.0 | 30.5 | 35.0 |
| Range | 21 - 44 | 20 - 62 | 23 - 37 | 30 - 54 | 21 - 54 | 25 - 39 | 20 - 62 |
| Height (cm) |  |  |  |  |  |  |  |
| n | 6 | 6 | 6 | 6 | 7 | 6 | 37 |
| Mean (SD) | 162.7 (8.09) | 171.7 (6.65) | 169.7 (9.03) | 170.8 (13.38) | 163.4 (7.52) | 174.2 (7.49) | 168.6 (9.35) |
| Median | 162.5 | 172.0 | 171.5 | 174.5 | 163.0 | 175.0 | 170.0 |
| Range | 154 - 176 | 162 - 182 | 156 - 181 | 148 - 187 | 155 - 176 | 162 - 183 | 148 - 187 |
| Weight (kg) |  |  |  |  |  |  |  |
| n | 6 | 6 | 6 | 6 | 7 | 6 | 37 |
| Mean (SD) | 66.17 (6.077) | 75.50 (10.900) | 67.88 (13.035) | 75.60 (11.601) | 66.84 (13.955) | 80.82 (7.759) | 71.99 (11.643) |
| Median | 63.35 | 73.40 | 70.75 | 73.65 | 67.40 | 78.40 | 73.10 |
| Range | 60.5 - 76.6 | 64.3 - 96.1 | 46.0 - 80.8 | 63.2 - 93.6 | 51.0 - 84.6 | 73.8 - 93.8 | 46.0 - 96.1 |
| Treatment Specification: A = One Spray of AM-301 Device per nostril; B = Two Sprays of AM-301 Device per nostril (with different spray angles); C = No Treatment; SD = standard deviation; BMI = Body mass index. | | | | | | | |

**Table S4 a-c**. Statistics for the efficacy analysis of Overall Change of TNSS (0 – 180 min) for **(A)** primary endpoint (ITT dataset), **(B)** primary endpoint (PP dataset, sensitivity analysis) and **(C)** Type 3 tests of fixed effects of ANCOVA model. Treatment Specification: Treatment A: One Spray of AM-301 device per nostril; Treatment B: Two Sprays of AM-301 device per nostril (with different spray angles); Treatment C: No treatment; D = A + B.

**a**

| **Treatment** | **N** | **Mean** | **SD** | **LS Mean (SE)** | **95% CI** | **ANCOVA**  **p value** |
| --- | --- | --- | --- | --- | --- | --- |
| **A** | 36 | 3.9 | 2.59 | 3.99 (0.452) | 3.08, 4.89 |  |
| **B** | 36 | 4.1 | 2.93 | 4.19 (0.452) | 3.29, 5.10 |  |
| **C** | 34 | 5.2 | 2.58 | 5.17 (0.460) | 4.25, 6.09 |  |
| **A vs. C*** |  |  |  | -1.18 (0.454) | -2.09, 0.27 | 0.0116 |
| **B vs. C*** |  |  |  | -0.98 (0.454) | -1.88, -0.07 | 0.0354 |
| **D vs. C*** |  |  |  | -1.08 (0.397) | -1.87, -0.28 | 0.0085 |

CI = Confidence Interval

**b**

| **Treatment** | **N** | **Mean** | **SD** | **LS Mean (SE)** | **95% CI** | **ANCOVA**  **p value** |
| --- | --- | --- | --- | --- | --- | --- |
| **A** | 32 | 3.8 | 2.72 | 3.81 (0.483) | 2.84, 4.78 |  |
| **B** | 32 | 4.1 | 2.93 | 4.15 (0.482) | 3.18, 5.12 |  |
| **C** | 32 | 4.9 | 2.31 | 4.93 (0.483) | 3.96, 5.90 |  |
| **A vs. C*** |  |  |  | -1.12 (0.468) | -2.05, -0.18 | 0.0202 |
| **B vs. C*** |  |  |  | -0.78 (0.464) | -1.71, 0.15 | 0.0989 |
| **D vs. C*** |  |  |  | -0.95 (0.404) | -1.76, -0.14 | 0.0223 |

CI = Confidence Interval

**c**

| **Effect** | **Num DF** | **Den DF** | **F Value** | **Pr > F** |
| --- | --- | --- | --- | --- |
| Base | 1 | 92.3 | 0.16 | 0.6876 |
| Sequence (ABC, BCA, CAB, ACB, CBA, BAC) | 5 | 29 | 0.73 | 0.6084 |
| Visit Number (3, 4 or 5) | 2 | 63.4 | 4.22 | 0.0190 |
| Treatment (A, B or C) | 2 | 63.5 | 3.79 | 0.0278 |

CI = Confidence Interval

**Table S5.** Difference of TNSS at Individual Time Points During House Dust Mite Challenge in the EEC (0-180 min) between AM-301 (D) and no treatment (C)

| Time Point | Treatment | N | Mean | SD | LS Mean (SE) | 95% CI | ANCOVA  p value |
| --- | --- | --- | --- | --- | --- | --- | --- |
| Pre EEC | A | 36 | 0.1 | 0.35 | 0.13 (0.070) | -0.01, 0.27 |  |
|  | B | 36 | 0.1 | 0.49 | 0.13 (0.070) | -0.01, 0.27 |  |
|  | C | 34 | 0.2 | 0.39 | 0.19 (0.071) | 0.04, 0.33 |  |
|  | D vs. C |  |  |  | -0.06 (0.069) | -0.19, 0.08 | 0.4165 |
| 20 min | A | 36 | 2.3 | 2.42 | 2.30 (0.417) | 1.47, 3.14 |  |
|  | B | 36 | 2.4 | 2.64 | 2.44 (0.418) | 1.60, 3.27 |  |
|  | C | 34 | 2.3 | 2.25 | 2.28 (0.427) | 1.43, 3.13 |  |
|  | D vs. C |  |  |  | 0.09 (0.415) | -0.74, 0.92 | 0.8309 |
| 40 min | A | 36 | 3.6 | 2.71 | 3.70 (0.487) | 2.73, 4.67 |  |
|  | B | 36 | 3.6 | 2.85 | 3.60 (0.488) | 2.63, 4.57 |  |
|  | C | 34 | 4.2 | 3.09 | 4.12 (0.500) | 3.13, 5.12 |  |
|  | D vs. C |  |  |  | -0.47 (0.524) | -1.51, 0.58 | 0.3735 |
| 60 min | A | 36 | 4.6 | 2.79 | 4.70 (0.498) | 3.71, 5.70 |  |
|  | B | 36 | 3.6 | 3.04 | 3.70 (0.499) | 2.71, 4.69 |  |
|  | C | 34 | 4.8 | 3.42 | 4.77 (0.511) | 3.75, 5.78 |  |
|  | D vs. C |  |  |  | -0.57 (0.533) | -1.63, 0.50 | 0.2915 |
| 80 min | A | 36 | 4.3 | 3.05 | 4.39 (0.493) | 3.40, 5.37 |  |
|  | B | 36 | 4.3 | 3.18 | 4.30 (0.494) | 3.32, 5.28 |  |
|  | C | 34 | 5.6 | 2.77 | 5.61 (0.505) | 4.60, 6.61 |  |
|  | D vs. C |  |  |  | -1.27 (0.500) | -2.26, -0.27 | 0.0137 |
| 100 min | A | 36 | 4.2 | 2.99 | 4.28 (0.524) | 3.23, 5.32 |  |
|  | B | 36 | 4.7 | 3.40 | 4.70 (0.518) | 3.67, 5.73 |  |
|  | C | 34 | 6.5 | 3.08 | 6.48 (0.529) | 5.42, 7.53 |  |
|  | D vs. C |  |  |  | -1.99 (0.498) | -2.98, -0.99 | 0.0002 |
| 120 min | A | 36 | 4.5 | 3.06 | 4.64 (0.563) | 3.51, 5.76 |  |
|  | B | 36 | 4.8 | 3.67 | 4.87 (0.558) | 3.75, 5.98 |  |
|  | C | 34 | 6.2 | 3.32 | 6.15 (0.569) | 5.02, 7.29 |  |
|  | D vs. C |  |  |  | -1.40 (0.519) | -2.44, -0.36 | 0.0089 |
| 140 min | A | 36 | 4.4 | 3.00 | 4.54 (0.545) | 3.46, 5.63 |  |
|  | B | 36 | 5.1 | 3.69 | 5.16 (0.539) | 4.08, 6.24 |  |
|  | C | 34 | 6.3 | 2.96 | 6.24 (0.551) | 5.14, 7.34 |  |
|  | D vs. C |  |  |  | -1.39 (0.534) | -2.46, -0.32 | 0.0114 |
| 160 min | A | 36 | 4.1 | 3.33 | 4.25 (0.587) | 3.07, 5.42 |  |
|  | B | 36 | 4.9 | 3.80 | 4.91 (0.582) | 3.74, 6.08 |  |
|  | C | 34 | 6.3 | 3.03 | 6.35 (0.591) | 5.17, 7.54 |  |
|  | D vs. C |  |  |  | -1.78 (0.485) | -2.74, -0.81 | 0.0005 |
| 180 min | A | 36 | 4.3 | 3.51 | 4.48 (0.616) | 3.25, 5.72 |  |
|  | B | 36 | 5.1 | 3.81 | 5.15 (0.611) | 3.93, 6.37 |  |
|  | C | 34 | 6.2 | 3.32 | 6.24 (0.621) | 5.00, 7.48 |  |
|  | D vs. C |  |  |  | -1.43 (0.523) | -2.47, -0.38 | 0.0082 |

ITT Population. CI = Confidence Interval, SD = Standard Deviation, LS Means from ANCOVA

**Table S6.** Overall change from baseline in individual nasal symptom scores (NSS)

| Individual Nasal Symptom | Treatment | N | Mean | SD | LS Mean (SE) | 95% CI | ANCOVA  p value |
| --- | --- | --- | --- | --- | --- | --- | --- |
| Itchy Nose | A | 36 | 1.1 | 0.79 | 1.09 (0.13) | 0.83, 1.35 |  |
|  | B | 36 | 1.1 | 0.83 | 1.12 (0.13) | 0.86, 1.38 |  |
|  | C | 34 | 1.4 | 0.65 | 1.38 (0.13) | 1.12, 1.64 |  |
|  | A vs. C* |  |  |  | -0.29 (0.12) | -0.54, -0.04 | 0.0224 |
|  | B vs. C* |  |  |  | -0.26 (0.12) | -0.50, -0.01 | 0.0412 |
|  | D vs. C* |  |  |  | -0.27 (0.11) | -0.49, -0.06 | 0.0138 |
| Nasal Congestion | A | 36 | 1.1 | 0.78 | 1.14 (0.13) | 0.88, 1.40 |  |
|  | B | 36 | 1.1 | 0.80 | 1.16 (0.13) | 0.90, 1.42 |  |
|  | C | 34 | 1.4 | 0.74 | 1.37 (0.13) | 1.10, 1.63 |  |
|  | A vs. C* |  |  |  | -0.22 (0.12) | -0.46, 0.01 | 0.0575 |
|  | B vs. C* |  |  |  | -0.21 (0.12) | -0.44, 0.03 | 0.0802 |
|  | D vs. C* |  |  |  | -0.22 (0.10) | -0.42, -0.01 | 0.0376 |
| Runny Nose | A | 36 | 1.0 | 0.72 | 1.07 (0.12) | 0.83, 1.31 |  |
|  | B | 36 | 1.1 | 0.73 | 1.12 (0.12) | 0.88, 1.35 |  |
|  | C | 34 | 1.4 | 0.72 | 1.40 (0.12) | 1.16, 1.64 |  |
|  | A vs. C* |  |  |  | -0.33 (0.12) | -0.57, -0.09 | 0.0079 |
|  | B vs. C* |  |  |  | -0.29 (0.12) | -0.53, -0.04 | 0.0209 |
|  | D vs. C* |  |  |  | -0.31 (0.11) | -0.52, -0.10 | 0.0047 |
| Sneezing | A | 36 | 0.7 | 0.69 | 0.69 (0.12) | 0.44, 0.94 |  |
|  | B | 36 | 0.8 | 0.79 | 0.80 (0.12) | 0.55, 1.05 |  |
|  | C | 34 | 1.0 | 0.77 | 1.01 (0.13) | 0.76, 1.26 |  |
|  | A vs. C* |  |  |  | -0.32 (0.13) | -0.59, -0.05 | 0.0209 |
|  | B vs. C* |  |  |  | -0.21 (0.13) | -0.48, 0.06 | 0.1256 |
|  | D vs. C* |  |  |  | -0.26 (0.12) | -0.50, -0.03 | 0.0283 |

ITT population. CI = Confidence Interval, SD = Standard Deviation, LS Means from ANCOVA

**TABLE S7.** Adverse Events (AEs) table.

| **System Organ Class**  **Preferred Term** | **AM-301**  **Treatment A**  **N=36**  **N (%) [E]** | **AM-301**  **Treatment B**  **N=37**  **N (%) [E]** | **No Treatment**  **N=37**  **N (%) [E]** | **Total**  **N=37**  **N (%) [E]** |
| --- | --- | --- | --- | --- |
| Subjects with at Least One AE | 4 (11.1) [4] | 0 (0) [0] | 1 (2.7) [1] | 5 (13.5) [5] |
| Immune System Disorder |  |  |  |  |
| Type I hypersensitivity | 1 (2.8) [1] | 0 (0) [0] | 0 (0) [0] | 1 (2.7) [1] |
| Investigations | 0 (0) [0] | 0 (0) [0] | 1 (2.7) [1] | 1 (2.7) [1] |
| SARS-CoV-2 test positive | 0 (0) [0] | 0 (0) [0] | 1 (2.7) [1] | 1 (2.7) [1] |
| Nervous system disorders | 2 (5.6) [2] | 0 (0) [0] | 0 (0) [0] | 2 (5.4) [2] |
| Headache | 1 (2.8) [1] | 0 (0) [0] | 0 (0) [0] | 1 (2.7) [1] |
| Taste disorder | 1 (2.8) [1]* | 0 (0) [0] | 0 (0) [0] | 1 (2.7) [1] |
| Respiratory thoracic & mediastinal disorders | 1 (2.8) [1] | 0 (0) [0] | 0 (0) [0] | 1 (2.7) [1] |
| Epistaxis | 1 (2.8) [1] | 0 (0) [0] | 0 (0) [0] | 1 (2.7) [1] |

N = number subjects, E = Number of events
